# Construction of an immune-related gene prognostic model for obese endometrial cancer patients based on bioinformatics analysis

**DOI:** 10.1016/j.heliyon.2024.e35488

**Published:** 2024-07-30

**Authors:** Yun Tong, Tao Zhu, Fei Xu, Wenjun Yang, Yakun Wang, Xianze Zhang, Xiujie Chen, Lei Liu

**Affiliations:** a Department of Pharmacogenomics, College of Bioinformatics Science and Technology, Harbin Medical University, Harbin, 150081, China; b Department of Pharmacy, Beidahuang Industry Group General Hospital, Harbin, 150088, China

**Keywords:** Endometrial cancer, Obesity, Tumor microenvironment, Immunity, Risk prognosis scores, Prognosis

## Abstract

**Background:**

The tumor microenvironment (TME) affected the prognosis of tumors. However, its effect on the outcomes of obese endometrial cancer (EC) patients had not been reported.

**Methods:**

This research performed a retrospective analysis of the transcriptome profiles and medical data of 503 EC patients. Immune scores were assessed by estimation algorithms. Cox and LASSO regression analyses were utilized to pinpoint key genes linked to prognosis, and the RPS was created to forecast the outcomes of obese EC patients. The relationship among genetic mutations and RPS was examined using CNV and somatic mutation information. ssGSEA and GSVA were employed to detect immune infiltration and immune pathway enrichment associated with key genes. The TIDE algorithm and GDSC database were utilized to forecast patients’ responses of patients to immunotherapy and chemotherapy, respectively. Finally, we employed the 'rms' R software package to construct the nomogram.

**Results:**

The prognosis of obese EC patients was associated with immune scores. Three key genes (EYA4, MBOAT2 and SCGB2A1) were identified. The risk prognosis score (RPS) for obese EC patients was established by risk stratification and prognostic prediction using prognostic genes. The higher the RPS, the worse the prognosis, and the more malignant the genomic alterations. The high RPS group had a significantly reduced proportion of most immune cells in comparison to the low RPS group. The high RPS group was linked to G2M, MYC and E2F related pathways such as cell proliferation, cell cycle and cell death. Cisplatin, tamoxifen and topotecan had a greater effect on the low RPS group. Notably, the nomogram had a good predictive ability.

**Conclusion:**

Our study designed a reliable RPS for obese EC patients to forecast their prognosis, immune aggressiveness, and responses to immunotherapy and drug treatments.

## Highlights

• Immune infiltration affected outcomes in patients with obesity and endometrial cancer (EC).
• Risk prognostic score (RPS) served as an independent predictor in EC patients with obesity.
• RPS model had the potential for better prognosis prediction, immunotherapy, and drug therapy in patients with obesity and EC.
• EC patients with obesity in high-RPS group had a more deleterious profile of clinicopathological and genomic aberrations.

## 1 Introduction

Endometrial cancer (EC) ranked high among global cancers [1]. According to the World Cancer Research Foundation, many cancers were associated with obesity as a significant risk factor, including EC. More than half of EC patients were related to obesity [2]. Obesity was considered an independent risk component [3,4]. When BMI increased by 5 kg/m^2^, the death risk of EC would increase by 1.59 times [5], and the mortality rate of morbidly obese patients was higher [[6], [7], [8], [9]]. Obesity was a chronic pro-inflammatory state [10]. Previous studies had found that increased chronic inflammation, increased visfatin and insulin resistance in obese patients were all mechanisms that advance the growth and death of EC [11]. The degree of circulation was augmented by inflammatory markers like tumor necrosis factor-α and C-reactive protein [12]. These inflammatory indicators might change the endometrial tumor microenvironment (TME) [13].

Increasing research showed that the TME was key to regulated tumor prognosis [14,15]. TME consisted of immune cells, inflammatory cells and extracellular matrix (ECM) [16,17]. The ECM had structural adhesion to tumor cells, mesenchymal cells, inflammatory cells and endothelial cells form a huge cell network, which provided paracrine factors for epithelial cells, thus promoting the progress of EC [18]. Moreover, TME can inhibit the immune system and consequently enhance the proliferation of malignant tumors. However, how obesity affected the prognosis, immune infiltration and immunotherapy of obese EC patients was still unclear and needs further study [19].

Therefore, we studied the TME in obese EC patients and assessed its role in forecasting through integrated study of multi-omics data. At the same time, we devised a prognostic prediction model in accordance with characteristic genes linked to immune infiltration. This model could forecast patients’ prognosis, the efficacy of immunotherapy, and the drug treatment response.

## 2 Materials and methods

### 2.1 Data acquisition and preliminary processing

RNA transcription data, clinical data, somatic mutation data, and copy number variation (CNV) data of EC patients were obtained from The Cancer Genome Atlas (TCGA) database. The IMvigor210 dataset was sourced from the R software package “IMvigor210CoreBiologies”. The GSE135222 cohort was acquired from the Gene Expression Omnibus (GEO) database. All data were cleaned of missing and null values, and only patients' data with survival information were retained. Patients with BMI over 30 were defined as obese.

### 2.2 Screening and construction of risk prognosis score (RPS) based on key genes of immune signature

The “ESTIMATE” algorithm was employed to evaluate the immune score of obese EC patients [20]. Differentially expressed genes (DEGs) were pinpointed utilizing the ‘limma’ R software package, with parameters set at p < 0.05 and |logFC|>1 [21]. The independent prognostic significance of DEGs was determined through Cox regression. Finally, the most predictive DEGs and their regression coefficients (β) were evaluated through Lasso regression. The risk prognosis score (RPS) was computed employing the subsequent formula: RPS = $\sum_{i=1}^{n}\left({Gene\;\exp}_{i}*\beta_{i}\right)$ . The area under curve (AUC) value and the efficacy of the RPS were measured by the “timeROC” R software package.

### 2.3 Modelling verification

Since we did not find any other EC data from other platforms to validate our model, but we found through previous studies that cervical cancer and ovarian cancer both occurred in the uterus and had a high consistency with EC [22], we chose cervical cancer and ovarian cancer data to validate our model. PD-1/PD-L1 was the key target of anti-cancer immunotherapy, and its mechanism of action had a lot of commonality in tumor immunotherapy [23,24]. At that time, clinical immunological agents were an effective means of EC treatment [25]. Since we did not find any EC immunotherapy data, we used data from patients with urothelial carcinoma [26] and advanced non-small cell lung cancer (NSCLC) [27] who underwent PD-1/PD-L1 treatment to verify our predicting effectiveness of immunotherapy.

### 2.4 Analysis of genetic alterations

The “maftools” software package [28] was applied to process somatic mutation data and identify the genes with the most mutation frequency. Genomic Identification of Significant Targets in Cancer (GISTIC) 2.0 [29] analyzed the CNV genes, and the gene deletion or amplification and GISTIC score were determined.

### 2.5 Immune cell estimation and gene set variation analysis (GSVA)

Single-sample gene set enrichment analysis (ssGSEA) method was used to calculate the percentage of 28 human immune cell categories in each sample [30]. The immune characteristics of each patient were established by gene set variation analysis (GSVA) using the “GSVA” R software package [31], determined the immune pathways related to the characteristic genes, focused on the analysis of carcinogenic signal pathways, and further carried out correlation analysis. The “corrgram” and “pheatmap” R software packages were utilized to draw relationship maps and heat maps.

### 2.6 Prediction of immunotherapy and drug treatment response

The Tumor Immune Dysfunction and Exclusion (TIDE) [32] algorithm was utilized to deduce the clinical response of obese EC patients to immune checkpoint therapy. The outcome of drug therapy in obese EC patients was calculated by Genomics Database Cancer Drug Sensitivity Genomics (GDSC). The “oncpredict” R software package [33] was utilized to estimate the semi-maximum inhibitory concentration (IC50) which can represent the drug reaction.

### 2.7 Construct nomogram model

Multivariate Cox regression was utilized to analyze whether RPS, diabetes, hypertension, grade and age were related factors affecting the survival of patients. The “rms” R software package [34] built nomogram [35], and evaluated and quantified the performance of nomogram by calibrating curves and decision curve analysis (DCA) curves.

### 2.8 Analysis of statistical data

R software (version 4.1.1) was applied in all analyses. Kaplan-Meier analysis was conducted using the “survival” R software package and the log-rank test The Spearman correlation coefficient by the “ggplot2″ software package was utilized to analyze correlations between RPS and immune signals. A p-value of less than 0.05 was deemed statistically significant.

## 3 Result

### 3.1 Clinical features of the studied subjects

**Fig. 1:**
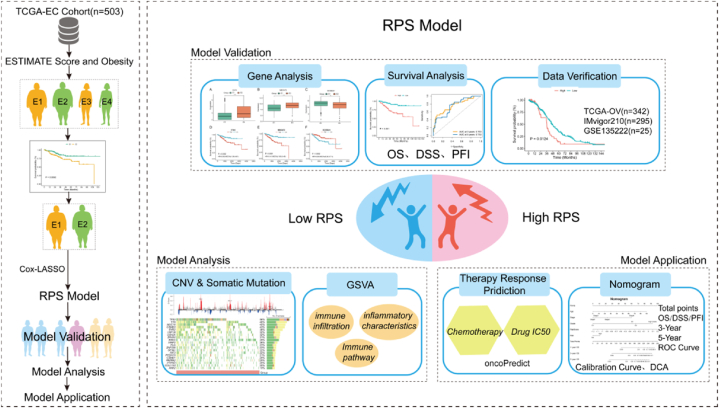
Study design flowchart.

We obtained data for 503 EC patients along with comprehensive clinical notes from the TCGA database to use as training datasets. We used the survival data from 342 ovarian cancer patients and 262 cervical cancer patients as our validation datasets. Supplementary Table S1 provided details on the clinical features of EC patients. We acquired data on 25 advanced NSCLC patients treated with PD-L1 and 295 patients with urothelial cancer from the GEO and the “IMvigor210CoreBiologies” R software package as the validation cohort of immunotherapy. Fig. 1 showed the research route.

### 3.2 The degree of immune infiltration had an effect on the prognosis of obese EC patients

**Fig. 2:**
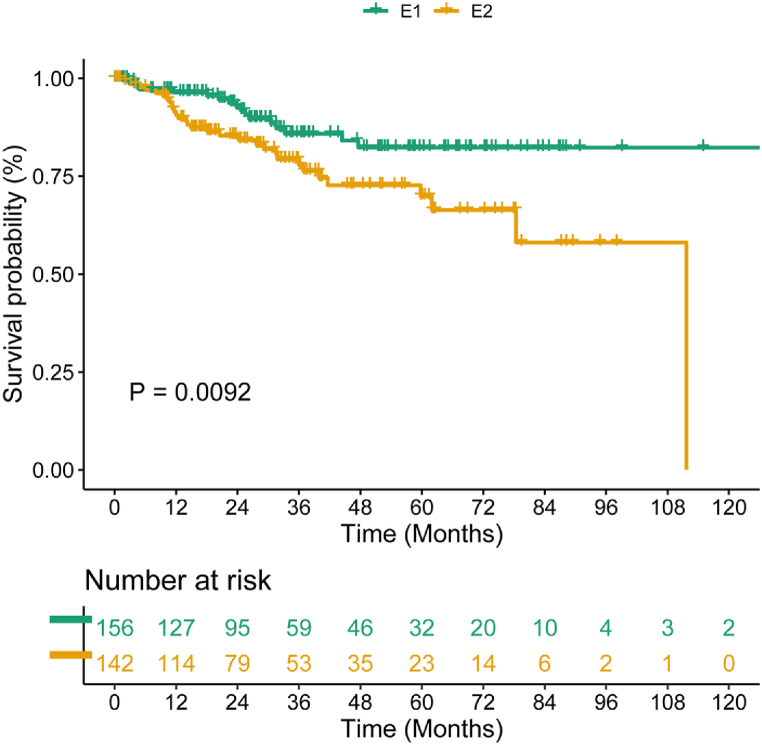
Comparison of overall survival (OS) prognosis between E1 and E2.

Earlier research has demonstrated that obesity was a predictive risk indicator for EC patients. However, according to our study on TCGA data, The survival rate was similar for obese patients (BMI at least 30) and non-obese patients (BMI under 30) (Supplementary Fig. S1A), and their immune scores also had no significant difference (Supplementary Fig. S1B). In addition, there was no variation in the BMI values of the high and low immune level cohorts (Supplementary Fig. S1C). As we considered, obesity and immune characteristics played a role in the prognosis of EC patients, which were important factors. In order to prove this hypothesis, We split the patients into four groups using the median of the immune score: obese group with high immune score (E1), obese group with low immune score (E2), non-obese group with high immune score (E3), and non-obese group with low immune score (E4). The prognostic analysis pointed out that only E2 had a poorer prognosis than E1 (Fig. 2, Log-rank, p = 0.0092), but the prognosis for E2, E3 and E4 did not differ significantly (Supplementary Figs. S1D–F). The results indicated that immune characteristics affected the obese EC patients’ prognosis, and therefore the prognostic risk of obese EC patients should be combined with obesity and immunity. Supplementary Table S2 presents the baseline characteristics of obese EC patients.

### 3.3 Identification of prognostic key genes based on immune characteristics

We compared the gene expression differences in obese EC patients to confirm the key genes that affected their prognosis. The results revealed that we identified 589 DEGs between E1 and E2. According to the Univariate Cox regression analysis, 171 DEGs demonstrated independent prognostic significance. Next, we utilized Lasso analysis to optimize the option of key genes. When we performed Lasso regression analysis, we randomized 1000 times, and the research results found that the number of genes was controlled between 3 and 18, and there were studies showing that when the minimum number of genes was taken, the model fitting effect was also very good, and the model was simpler [36]. Therefore, we chose the optimal lambda value (Supplementary Figs. S2A–B), obtained three key genes along with their respective coefficients (Supplementary Fig. S2C), and derived from these 3 key genes, we established the RPS model to foresee the obese EC patients' prognosis: RPS = (0.02965*EYA4)+(−0.06738*SCGB2A1)+(0.00616*MBOAT2). The formula employs Fragments Per Kilobase Million (FPKM) as the gene expression format. We categorized obese EC patients into high-RPS and low-RPS groups based on the median RPS. According to the patients' living state, the tripartite graph indicated that the RPS was substantially higher in the death cohort than in the survival cohort (Supplementary Fig. S2D), which demonstrated that the RPS model created in this study could effectively forecast the patient's prognosis.

**Fig. 3:**
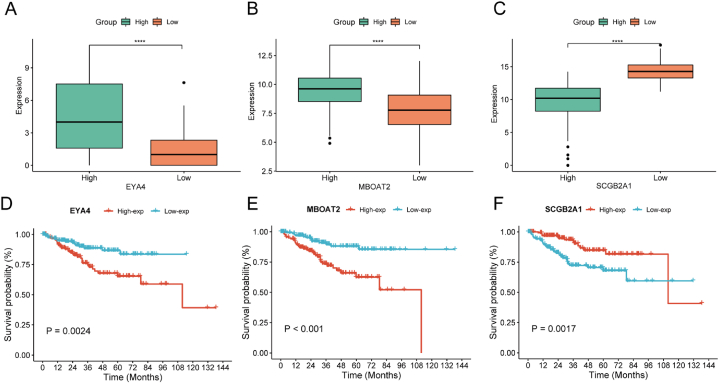
Identification of prognostic characteristic genes. (A–C) The expression differences of EYA4, MBOAT2, and SCGB2A1 among the high and low RPS groups were compared. (D–F) Kaplan-Meier diagram showed different prognoses based on the expression difference of EYA4, MBOAT2 and SCGB2A1. ****p < 0.0001.

We investigated the differences in expression of these three key genes comparing the tow groups and their prognostic implications based on their expression levels. The expression levels of EYA4 and MBOAT2 were higher in the high-RPS group, while the low-RPS group had higher expression level of SCGB2A1 (Wilcoxon, p < 0.0001, Fig. 3A–C). The high expression of EYA4 (Log-rank, p = 0.002) and MBOAT2 (Log-rank, p < 0.001) were linked to poor prognosis (Fig. 3D–E), while the high expression of SCGB2A1 (Log-rank, p = 0.002, Fig. 3F) was an indicator of favorable prognosis. This pointed to EYA4 and MBOAT2 were the risk-associated genes for the obese EC patients' prognosis, and SCGB2A1 acted as a protective gene in the obese EC patients’ prognosis.

### 3.4 The RPS we developed effectively predicts patient prognosis

**Fig. 4:**
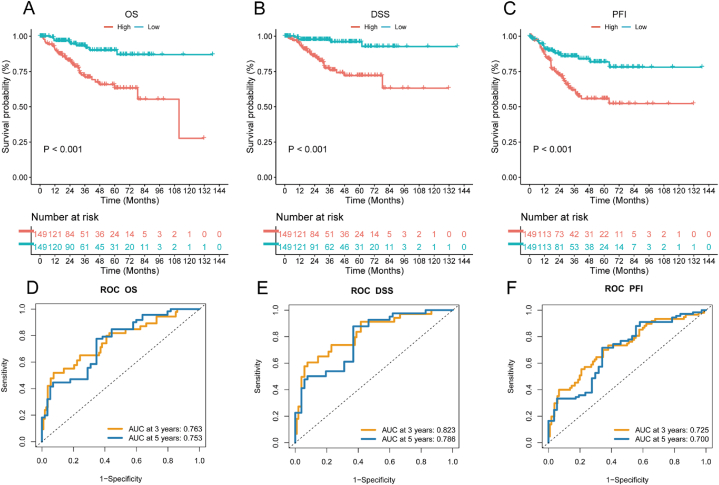
Evaluating the RPS. (A–C) Comparison of OS, DSS and PFI outcomes comparing the high-RPS group with the low-RPS group. (D-F) Receiver operating characteristic (ROC) curve and AUC value of RPS.

We evaluated the survival outcomes of the high-RPS group and low-RPS group. The results indicated significant differences in Fig. 4A–C (Log-rank, p < 0.001). The overall survival (OS), progression free interval (PFI) and disease specific survival (DSS) were significantly shorter in the high-RPS group (Log-rank, p < 0.001). The RPS forecasted the outcomes of obese EC patients, with AUC values for OS being 0.763 and 0.753, for DSS being 0.823 and 0.786, and for PFI being 0.725 and 0.700 (Fig. 4D–F). These results demonstrated that the RPS was effective in forecasting the outcomes of obese EC patients.

### 3.5 Verification of external data of RPS

Due to literature indicating that cervical cancer and ovarian cancer both occur in the uterus and have a high degree of consistency with EC, our data analysis revealed that ovarian cancer was more closely related to EC (Wilcoxon, P < 0.0001, Supplementary Figs. S3A–F and Supplementary Tables S5–S7). Therefore, we selected data from ovarian cancer patients for model validation, calculated the patients' RPS, and categorized them into high-RPS and low-RPS groups. It was observed that individuals in the high RPS group exhibited poorer prognostic outcomes (Log-rank, p < 0.05, Supplementary Fig. S4A). This suggested that RPS was effective in predicting patient outcomes. To validate the predictive capability of RPS regarding patient outcomes after immunotherapy, we selected the urothelial carcinoma dataset (IMvigor210) and the advanced NSCLC dataset treated with anti-PD-L1 (GSE135222) as the validation datasets. According to the RPS calculation results, we split the patients receiving immunotherapy in the GSE135222 and IMvigor210 into high-RPS and low-RPS groups. In GSE135222 (Supplementary Fig. S4B) and IMvigor210 (Supplementary Fig. S4C), the low-RPS group showed a notably extended OS. Considering the patients' reactions to immunotherapy, we divided the IMvigor210 cohort patients into stable disease (SD), progressive disease (PD), partial remission (PR) and complete remission (CR). The results of IMvigor210 showed that the ratio of SD/PD was higher in the high-RPS group patients, and CR/PR was more common in the low-RPS group patients (Supplementary Fig. S4D). The tow groups showed a significant variation in the response category (PR, CR, SD and PD) (Wilcoxon, p < 0.001, Supplementary Fig. S4E).

### 3.6 RPS was related to genomic aberration feature

**Fig. 5:**
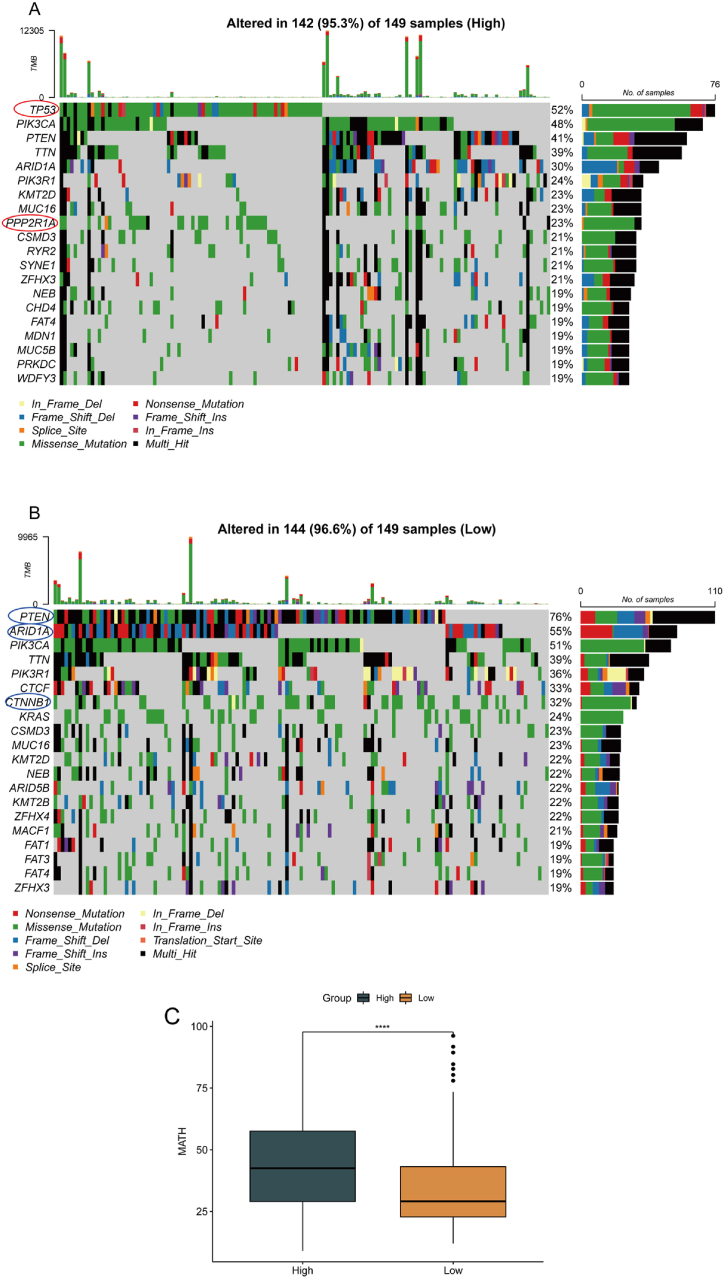
Analysis of somatic gene mutation. (A) Somatic gene mutation spectrum of high-RPS group. (B) Somatic gene mutation spectrum of low-RPS group. (C) Comparison of MATH values in tow groups. ****p < 0.0001.

Generally speaking, somatic mutations and CNV could lead to changes in the biological features of tumor cells, which could affect the growth, invasion, metastasis, immune escape and drug sensitivity of tumors, and thus lead to different prognostic outcomes [37]. We examined the gene mutation level of patients in the high-RPS and the low-RPS group, and showed the leading 20 genes with mutation rates in each in each cohort (Fig. 5A–B). 142 (95.3 %) individuals in the high-RPS group and 143 (95.97 %) individuals in the low-RPS group had somatic mutation. The mutation frequencies of PTEN, ARID1A and CTNNB1 genes in the low-RPS group were markedly higher (PTEN:76 % vs. 41 %; ARID1A:55 % vs.30 %; CTNNB1: 32 % vs. 0 %), while the high-RPS cohort displayed markedly higher mutation frequencies of TP53 and 2RP1A genes (TP53: 53 % vs. 0 %; 2RP1A: 23 % vs. 0 %) (Fig. 5A–B and Supplementary Fig. S5). TP53 was a cancer driver gene among the five genes with marked distinctions across the tow groups [38]. Furthermore, we applied the mutant allele tumor heterogeneity (MATH) algorithm to analyze tumor heterogeneity of the tow groups. MATH assessed genetic heterogeneity in tumors using all exome sequencing of tumors and their corresponding normal DNA [39]. The analysis demonstrated that the MATH value for the high-RPS group was notably higher (P < 0.0001). A higher MATH score indicated a higher proportion of subclonal mutations, which might make the tumor more aggressive. This pointed to greater heterogeneity in the tumors of the high-RPS group [40] (Fig. 5C, Wilcoxon, P < 0.05).

In the analysis of gene copy number variation, we found significant focal amplification and deletion in both high-RPS and low-RPS groups. The G value (representing the frequency of mutation of the gene in all samples) was markedly lower in the low-RPS group (Supplementary Fig. S6A, red and blue areas both indicated that the P value was less than 0.05). Relative to the low-RPS group, the high-RPS group displayed a focal deletion peak at 19p13.3 (AES, AMH, CSNK1G2), and several important amplification regions including 3q26.2 (MECOM, TERC, ACTRT3), 8q24.21 (MYC, CASC11) and 19q12 (CCNE1) (Supplementary Fig. S6B). Relevant literature showed that MECOM and MYC were oncogenes; the high occurrence rate of TERC mutation and the increase of CNV of TERC gene were related to the malignancy of tumors [41]; CASC11 acted as an oncogene in several cancer types [42]; the amplification of CCNE1 gene was more frequent in G3 than G2 in grade grading of endometrial carcinoma [43].

### 3.7 Immune correlation analysis and pathway enrichment analysis of RPS

**Fig. 6:**
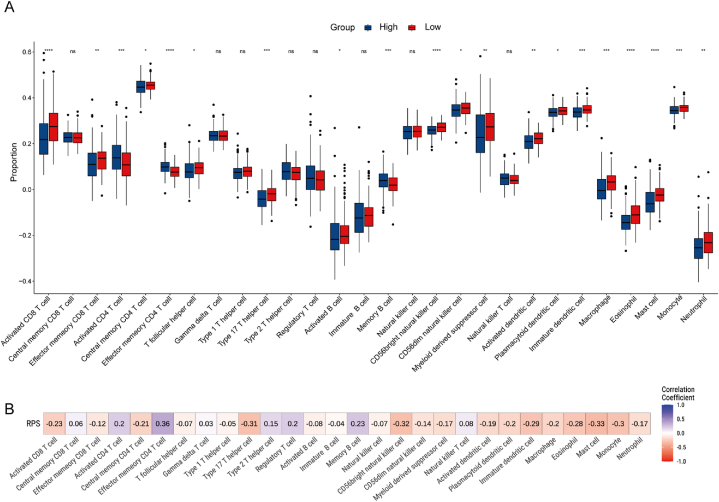
Immune cell infiltration characteristics of RPS. (A) Comparing the 28 immune cell components in the tow groups among obese EC patients. (B) Associations of RPS with the comparative abundance of 28 immune cells. ns p > 0.05,*p < 0.05, **p < 0.01, ***p < 0.001, ****p < 0.0001.

These findings suggested that the RPS derived from immune signature genes could prognosticate obese EC patients. Next, to further elucidate the association of RPS with the immune landscape, we utilized ssGSEA to assess the comparative abundance of the 28 immune cells. Specifically, in the high-RPS group, the penetration of activated B cells, effector memory CD8 T cells, T follicular helper cells, Th17 cells, activated dendritic cells, plasmacytoid dendritic cells, immature dendritic cells, macrophages, eosinophils, mast cells monocytes and neutrophils were significantly reduced (Wilcoxon, p < 0.05, Fig. 6A). Correlation analysis showed that RPS had a negative correlation the infiltration levels of most immune-activated, anti-tumor functional cells (such as activated CD8 T cells, CD56 bright natural killer cells and central memory CD4 T cells) and immune-suppressive, pro-tumor functional cells (such as CD56 dim natural killer cells, immature dendritic cells and macrophages) (Fig. 6B, Supplementary Table S3). Supplementary Fig. S7 showed the distribution characteristics of the top 10 immune cells related to the RPS, as well as the clinical features of different subgroups. The aforementioned findings additionally validated that RPS was inversely related to the immune cell infiltration rate in the TME of obese EC patients.

To investigate the underlying mechanisms connecting RPS and tumorigenesis, we performed GSVA detection in the tow group, and further elucidated the molecular mechanisms of the two groups' differences by HALLMARK gene set enrichment analysis. Nine hallmark pathways had markedly higher scores in the high-RPS group, with the majority linked to known oncogenic pathways [[44], [45], [46]] (Supplementary Fig. S8). Then we evaluated the predictive significance of the enhanced hallmark pathways, and survival rates varied markedly across the high and low enrichment score groups, such as G2M_CHECKPOINT, MYC_TARGETS_V2 and E2F_TARGETS (Log-rank, p < 0.05, Supplementary Fig. S9). The pathways were linked to multiple aspects such as cell proliferation, metabolism, cell cycle and cell death [47]. In summary, RPS was closely related to oncogenic pathways.

### 3.8 Correlation analysis of RPS with immunotherapy and chemotherapy response

**Fig. 7:**
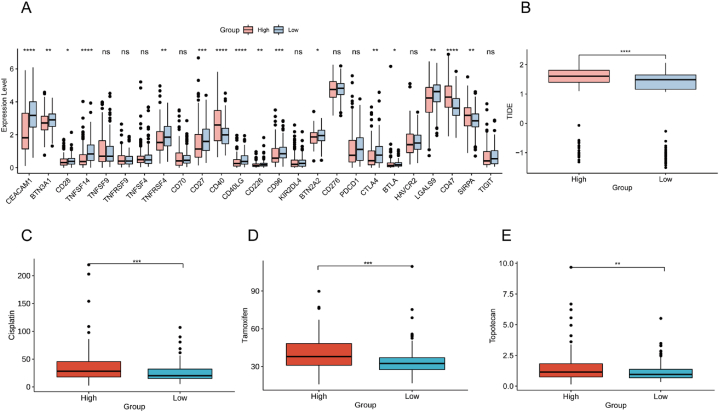
Application of RPS in immunotherapy prediction. (A) Expression of immune system checkpoint genes in the tow groups; (B) TIDE scores' distribution in obese EC patients; (C–E) IC50 estimates of cisplatin, tamoxifen and topotecan in the tow groups. ns p > 0.05,*p < 0.05, **p < 0.01, ***p < 0.001, ****p < 0.0001.

Immunotherapy was a key factor in tumor treatment, and the outcome of immunotherapy was strongly linked to the patients' prognosis. The main system of tumor immune evasion was the immune system checkpoint pathway [48]. To evaluate the ability of RPS as a biomarker for predicting the clinical response of immune checkpoint blockers, we examined the expression and drug sensitivity differences of different RPS groups in immune checkpoints. In Fig. 7A, our findings showed that the expression of most immune checkpoints had a strong association with the RPS (Wilcoxon, p < 0.05). These findings showed that the RPS had great potential in forecasting the reaction to immunotherapy in obese EC patients. Subsequently, we applied the TIDE algorithm to forecast the immunotherapy response of immune checkpoint inhibition in the data set of obese EC patients. The high-RPS group exhibited elevated TIDE scores, showing that the high-RPS group had a poorer reaction to immunotherapy (Wilcoxon, P < 0.0001, Fig. 7B). Next, we selected three drugs suitable for treating EC from 198 drugs. According to the results of drug sensitivity study, the IC50 values of cisplatin, tamoxifen and topotecan in the low RPS group were significantly lower (Wilcoxon, p < 0.01, Fig. 7C–E), indicating that these three drugs were more suitable for the low RPS group.

### 3.9 Nomogram predicts the survival of obese EC patients

**Fig. 8:**
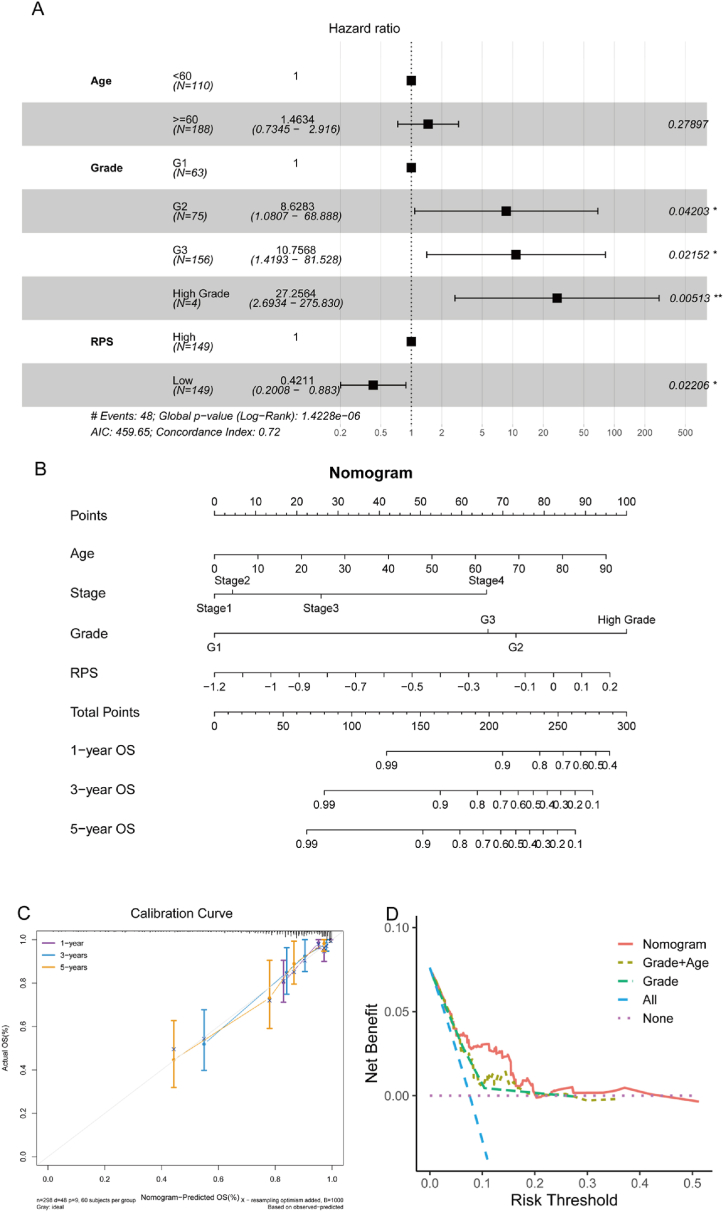
The nomogram predicted the obese EC patients' OS. (A) Plot of the outcomes of multivariate Cox regression analysis. (B) Nomogram for forecasting the 1-, 3- and 5-year overall survival rate. (C) Calibration curve of pr1edicted and observed OS rate. (D) Decision curve of net income comparison of nomogram.

To ascertain whether RPS was an independent predictive feature for obese EC patients, we performed single-variable and multiple-variable Cox regression analyses on RPS, age and grade. The results indicated that grade and RPS were independent predictors of obese EC patients' survival (Fig. 8A, Supplementary Table S4). To improve the prognostic power of RPS, we built a nomogram that integrated clinical data, which could more accurately forecast the outcome of obese EC patients (Fig. 8B). The calibration analysis revealed that the prediction curves of 3-and 5-year survival rates were very nearly the perfect performance (45-degree line), which implied that the nomogram model had a very great accuracy (Fig. 8C), and the integrated nomogram obtained a greater net benefit than the clinical features (Fig. 8D). Moreover, we also studied the prognostic prediction ability of the nomogram, and discover that the nomogram was strongly associated with OS, PFI and DSS (Log-rank, p < 0.01, Supplementary Figs. S10A–C). The AUC scores for 3-year and 5-year were between 0.816 and 0.798 for OS, 0.865 and 0.825 for DSS, and 0.757 and 0.736 for PFI (Supplementary Figs. S10D–F). Therefore, the nomogram had a high predictive ability for obese EC patients’ prognosis.

## 4 Discussion

In the past decade, the rapid development of genomic research had changed the traditional understanding of cancer and promoted the identification of new prognostic markers [49]. Studies showed that the increase in EC incidence was closely related to the prevalence of obesity and the associated hormonal imbalance [50]. It was estimated that 11 % of female cancers were linked to obesity and overweight as contributing risk factors, and the hormones and adipokines related to the general changes of obesity had a great impact on the growth of cancer [51]. Obesity also mediated the dysfunction of the body's microenvironment through components such as microbes, metabolites, growth factors and immune cells, thus accelerating the occurrence and development of tumors.

With the improvement in living standards, the proportion of obese EC patients increased year by year, and this group of people showed some specificity in many aspects such as immunotherapy effect and prognosis [52]. However, there was still a lack of specific markers that could reflect the immunological response and obese EC patients' prognosis. We targeted the prognosis, immune infiltration and reaction to pharmacotherapy of obese EC patients, established the RPS, and developed a predictive nomogram model using clinical data. At the same time, we also discussed the role of the TIME on the obese EC patients’ prognosis. We computed the immune score of the patients. The outcomes showed that the immune score was linked with the patients' survival rate, and a low immune score was associated with a worse prognosis. The biggest highlight of this study was the construction of RPS, which was composed of three genes: EYA4, MBOAT2 and SCGB2A1, and their coefficients.

EYA transcription coactivator and phosphatase 4 (EYA4) interacted with PI3K/AKT, Wnt/GSK-3β and various signal proteins of cell cycle pathway, and played its role in promoting or inhibiting cancer. Wnt/GSK-3β pathway was considered to have negative effects on lipogenesis and obesity. PI3K/AKT was a signal pathway in obesity-related pathogenesis [53]. In our research, the high expression of EYA4 indicated that the OS of obese EC patients was worse, suggesting that EYA4 might be an unfavorable prognostic marker for obese EC patients.

Membrane-bound O-acyltransferase domain containing 2 (MBOAT2) gene in EC had not been reported, but some research results showed that MBOAT2 was a likely proto-oncogene in pancreatic cancer, which could foresee predict the prognosis of pancreatic cancer patients [54]. MBOAT2 was one of the DHA-induced lipid metabolism genes, and its expression was closely associated with the process of lipid metabolism and fatty acid changes [55]. In our study, the high expression of MBOAT2 also showed poor prognosis in obese EC patients, suggesting that MBOAT2 might also be an unfavorable prognostic marker.

Secretoglobin family 2A member 1 (SCGB2A1) was mainly expressed in the breast, uterus and salivary gland [56]. The increase of its expression could reduce the aggressive behavior of cancer cells, lower the risk of disease recurrence and growth, and be linked to the good prognosis of ovarian cancer [57]. In addition, Dieters-Castator et al. [58] demonstrated that SCGB2A1, was markedly related to a good prognosis for EC, which was also consistent with the findings presented here. Genes in the same family had similar biological functions [59]. Club cell secretory protein-16 (CC16) was transcribed by SCGB1A1. The level of CC16 was inversely related to BMI, indicating that obesity itself would affect the expression of CC16 protein in bronchioles [60].

We established the RPS based on the above characteristic genes. We discovered that the low expression of EYA4 and MBOAT2 genes indicated a poor prognosis related to the high immune score group patients, whereas the high expression of SCGB2A1 gene indicated a better prognosis. The prognosis for obese EC patients in the high-RPS group was worse. RPS was identified as a factor with a unique prognostic value.

Since the nomogram had a high clinical utility, we combined the established RPS with the patient's age and grade, and developed a nomogram to foresee the obese EC patients' prognosis. According to the RPS, we split the patients into high-RPS and low-RPS group depending on their prognosis. Through gene mutation analysis, we found that the high-RPS group exhibited lower mutation frequencies of PTEN, ARID1A, and CTNNB1 genes. We also found important amplification regions (MECOM, TERC, ACTRT3, MYC, CASC11, CCNE1) and deletion peaks (AES, AMH, CSNK1G2) of various oncogenes in patients in the high-RPS group. Different gene mutations could affect the growth, invasion, metastasis, immune escape and drug sensitivity of tumors, and thus leading to different prognostic outcomes. Therefore, more basic research and clinical trials were needed to accurately assess the association between gene mutations and prognosis.

Our study further explored the connection between TME and RPS. Patients with high-RPS group and low-RPS group exhibited distinct traits of immune cell penetration. The osmosis status of memory B cells and activated CD4 T cells in high-RPS were higher, while those of activated B cells and effector memory CD8 T cells in patients with low-RPS were increased. CD56 bright cells were a functional subgroup of NK cells, which played an immunomodulatory role [61]. Compared with CD56dim NK cells, the infusion of IL-15 markedly expanded the subset of NK CD56bright cells [62]. Combined with our research, we believed that NK CD56bright cells played a vital part in the immunity of obese EC patients, and their infiltration can improve the prognosis. Moreover, Some research had indicated that obesity was the main risk factor of 11 kinds of cancers, however, the influence of obesity on tumor immune reaction was largely unknown [63]. Obesity can promote tumor growth, reduce infiltration of CD8 T cell and proliferate tumor function. Impaired CD8T cell infiltration and effector function were related to amino acid metabolism and damaged chemokine expression in obese patients [19]. According to our research results, obesity was a major influencing factor for the prognosis of EC, and it significantly affected the immune function of the patients.

Previous studies had found that increased chronic inflammation and adipokine were how obesity promoted EC [11]. According to our study results, simple obesity did not greatly influence the prognosis of EC, and it needed to be comprehensively analyzed with the immune status to have a significant impact, which also suggested that Obesity had a comprehensive effect on EC patients' prognosis, but the level of impact might need a certain degree of excess. Only when the degree of obesity had a corresponding impact on the patient's immune status, such as greater infiltration levels of memory B cells and activated CD4 T cells, would it greatly influences the patient's prognosis. Some studies showed that the increase in BMI in EC patients was tied to the decrease of CD8 and PD-L1 expression, and one reason could be that the tumor mutation burden was low [64]. Higher BMI patients tended to be microsatellite stable, resulting in lower immune cell recognition ability and less expression of neoantigens. In addition, immune cell infiltration was boosted by weight loss surgery, suggesting that obesity directly affected the anti-tumor immunity of EC [65]. These research emphasized a new pathway through which obesity could enhance tumor development, which had broad implications for tumor treatment. How obesity affected the immunotherapy efficacy of obesity-related cancers (such as EC) was not clear, and more studies were needed to elaborate in detail [19]. Therefore, our study suggested that we should pay more attention to obese EC patients with high immune status and poor prognosis, and choose appropriate immunotherapy drugs to prolong their survival.

Of course, This project has some limitations. First, the project requires patients with EC to have expression profile data information, weight-related information, gene mutation information, CNV information, DNA methylation data and survival data at the same time. Therefore, the number of samples that meet the conditions from the existing public data resources (such as TCGA and GEO) was very limited, and the small sample size may have some impact on the accuracy of the research results. Second, this project lacks direct in vivo or in vitro research for validation. Finally, the specific mechanisms of the three immune characteristic key genes EYA4, SCGB2A1 and MBOAT2 on the EC obese patients need to be further explored and studied in more detail. The future project research will further strive to improve and make up for the above limitations, and also investigate the variations in chemotherapy outcomes more deeply and Immunosuppressive therapies among obese versus non-obese EC patients.

## 5 Conclusion

To summarize, the RPS was developed to forecast the patients’ prognosis with obese EC, and the possible prognosis-related mechanisms and immunological characteristics of EC obese patients were analyzed from the immune level. At the same time, based on the RPS, age and disease grading, a visual survival plot model was constructed, which can make more precise forecasts of the survival of EC obese patients in clinical practice. We very much hope that the RPS can enable EC obese patients to receive more accurate treatment and further research on immunotherapy, and also hope that the RPS can also be applied to forecast the prognosis for patients with different gynecological tumors, and provide patients with more accurate treatment guidance.

## Funding statement

This work was supported by the 10.13039/501100001809National Natural Science Foundation of China (Grant Nos. 61971166).

## Data availability statement

The data obtained and analyzed in this study are accessible in the TCGA repository (https://portal.gdc.cancer.gov/) and GEO database (https://www.ncbi.nlm.nih.gov/geo/). Data of obese patients with EC (TCGA-UCEC) were obtained from TCGA database, and GSE135222 was obtained from GEO database. The respective institutional review boards approved all those previous studies.

## CRediT authorship contribution statement

**Yun Tong:** Writing – original draft, Methodology, Conceptualization. **Tao Zhu:** Writing – review & editing. **Fei Xu:** Methodology, Conceptualization. **Wenjun Yang:** Software, Methodology, Data curation. **Yakun Wang:** Formal analysis. **Xianze Zhang:** Formal analysis. **Xiujie Chen:** Writing – review & editing. **Lei Liu:** Writing – review & editing, Supervision.

## Declaration of competing interest

The authors declare the following financial interests/personal relationships which may be considered as potential competing interests:Xiujie Chen reports financial support was provided by 10.13039/501100001809National Natural Science Foundation of China. If there are other authors, they declare that they have no known competing financial interests or personal relationships that could have appeared to influence the work reported in this paper.
