# Supplementary material for: Construction of an immune-related gene prognostic model for obese endometrial cancer patients based on bioinformatics analysis
Source: Heliyon. 2024 Jul 30;10(15):e35488. doi: 10.1016/j.heliyon.2024.e35488 (PMC11336703; doi:10.1016/j.heliyon.2024.e35488)
Supplement: Multimedia component 1 [file mmc1.docx]

**SUPPLEMENTARY INFORMATION**

**Construction of an immune-related gene prognostic model for obese endometrial cancer patients based on bioinformatics analysis**

Yun Tong^a,1^, Tao Zhu^b,1^, Fei Xu^a^, Wenjun Yang^a^, Yakun Wang^a^, Xianze Zhang^a^, Xiujie Chen^a,^**, Lei Liu^a,^*

^a^ Department of Pharmacogenomics, College of Bioinformatics Science and Technology, Harbin Medical University, Harbin 150081, China.

^b^ Department of pharmacy, Beidahuang Industry Group General Hospital, Harbin 150088, China.

* Corresponding author：

Lei Liu, liulei@ems.hrbmu.edu.cn, 86+13796821728

** Corresponding author：

Xiujie Chen, [chenxiujie@ems.hrbmu.edu.cn,](mailto:chenxiujie@ems.hrbmu.edu.cn,) 86+13945097808

^1^ These authors contributed equally to this work and should be considered co-first authors.

**Supplementary Figures：**

Figure S1. Comparative analysis of obese and non-obese patients with endometrial cancer.

Figure S2. Identify signature genes.

Figure S3. Analysis of key gene correlations in three types of cancer.

Figure S4. Validation of RPS.

Figure S5. Comparison of gene mutation frequency between high-RPS and low-RPS groups.

Figure S6. Analysis of gene copy number variation.

Figure S7. Heatmap of the RPS relationship with the top 10 immune cells.

Figure S8. The biological pathways had significant differences between high-RPS and low-RPS groups in GSVA.

Figure S9. Kaplan-Meier survival plots showing the significant correlations between the OS and GSVA scores of typical oncogenic hallmark pathways.

Figure S10. Evaluation of the nomogram.

**Supplementary Table**

Table S1.The characteristics of all included patients.

Table S2.Baseline data of UCEC patients with obesity.

Table S3. Correlation between RPS and immune cell infiltration.

Table S4. Univariate Cox regression analysis of RPS.

Table S5. Correlation analysis among key genes in endometrial cancer.

Table S6. Correlation analysis among key genes in ovarian cancer.

Table S7. Correlation analysis among key genes in cervical cancer.

**Supplementary Figures**


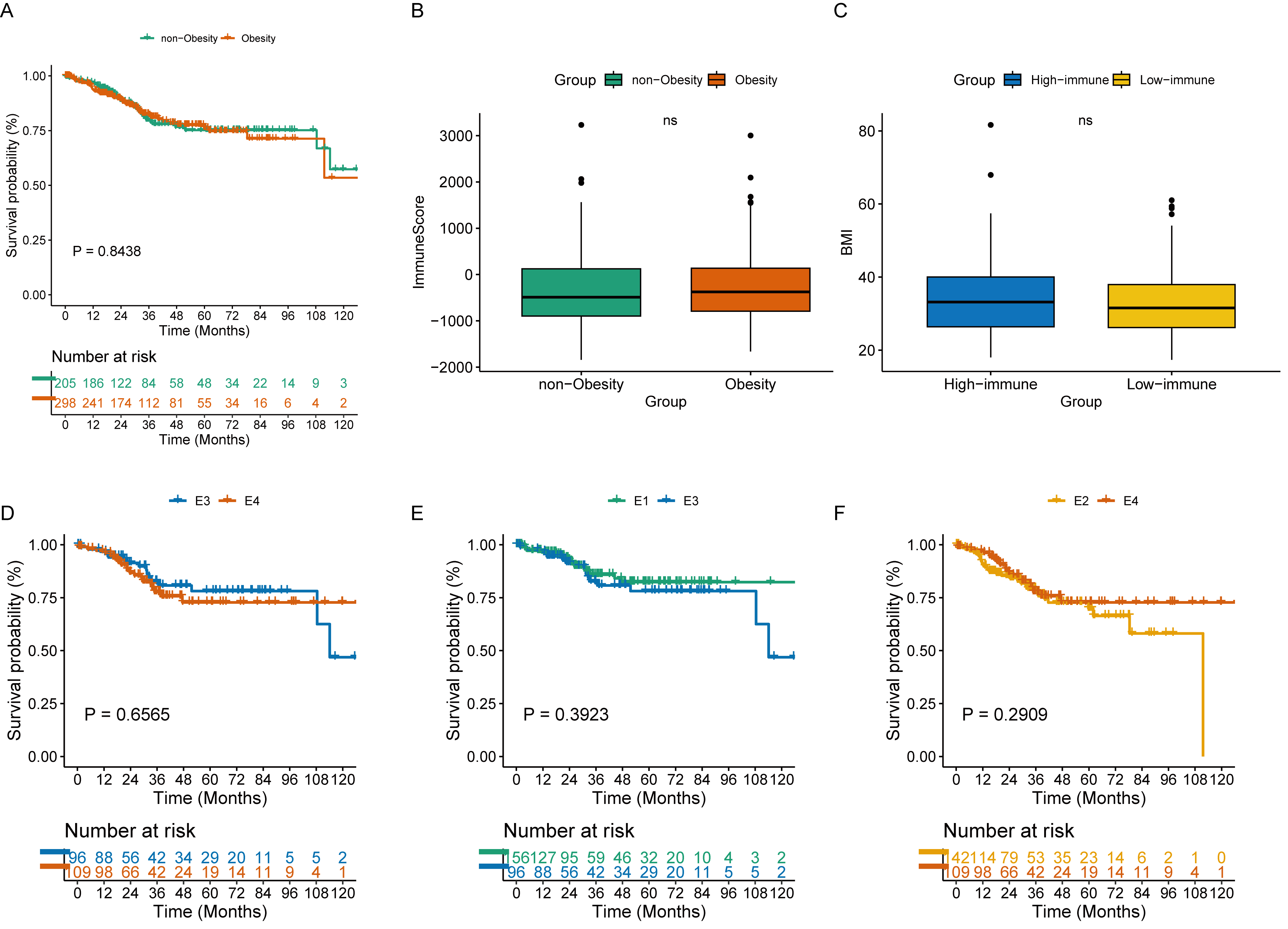


**Figure S1. Comparative analysis of obese and non-obese patients with endometrial cancer.** **(A)** Survival analysis of obese patients and non-obese patients. **(B)** Comparison of immune score box chart between obese patients and non-obese patients. **(C)** Comparison of box plots of BMI values between high immune score and low immune score groups. **(D)** Survival analysis of non-obese group with high immune score (E3) and non-obese group with low immune score (E4). **(E)** Survival analysis of non-obese group with high immune score (E3) and obese group with high immune score (E1). **(F)** Survival analysis of obese group with low immune score (E2) and non-obese group with low immune score (E4).


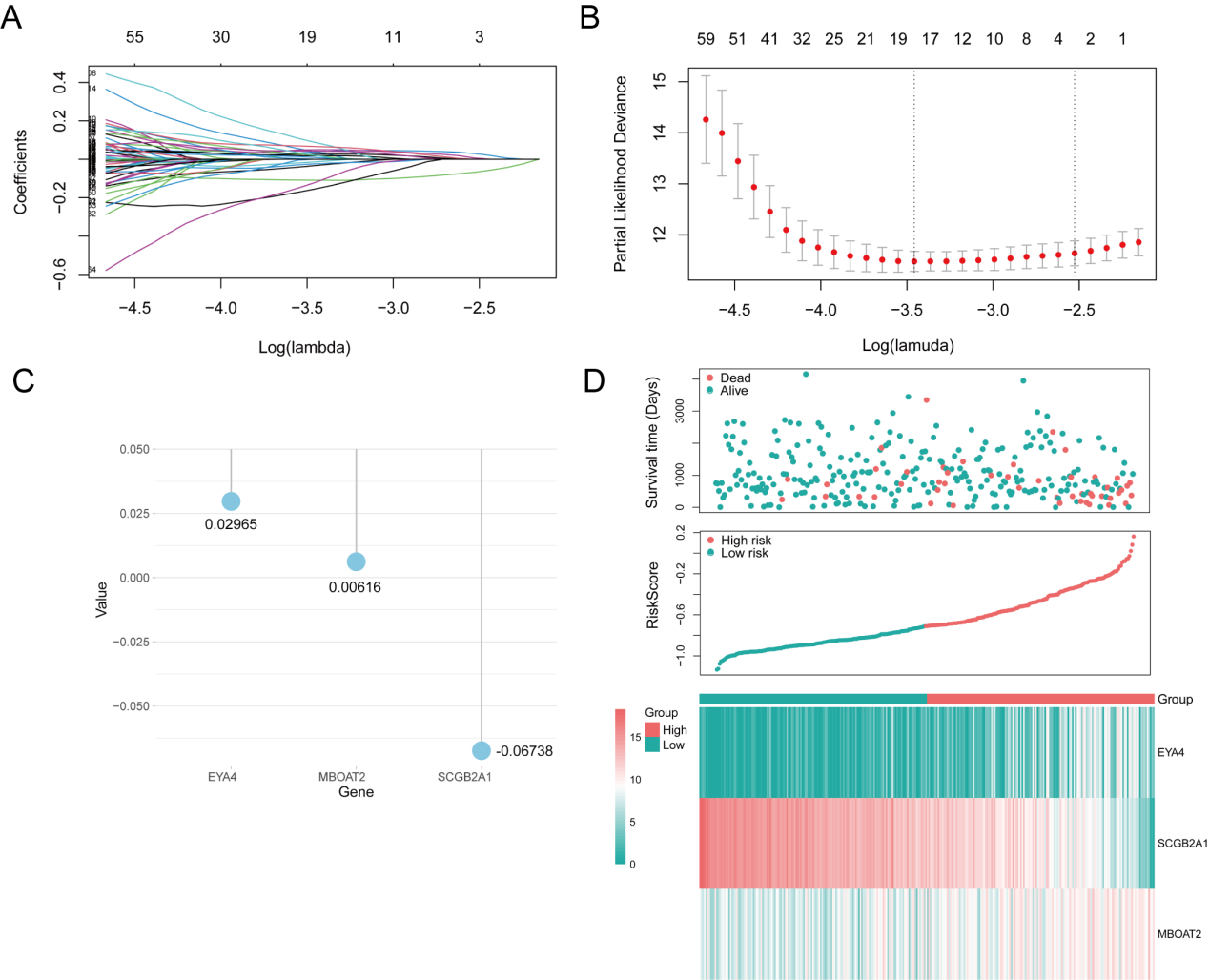


**Figure S2. Identify signature genes. (A)** LASSO coefficient of 171 differentially expressed genes with independent prognostic value. **(B)** Cross-validation plot of lasso regression (dotted line indicates optimal log lambda value). **(C)** Lasso coefficients of key genes. **(D)**Tripartite graph of risk factors.


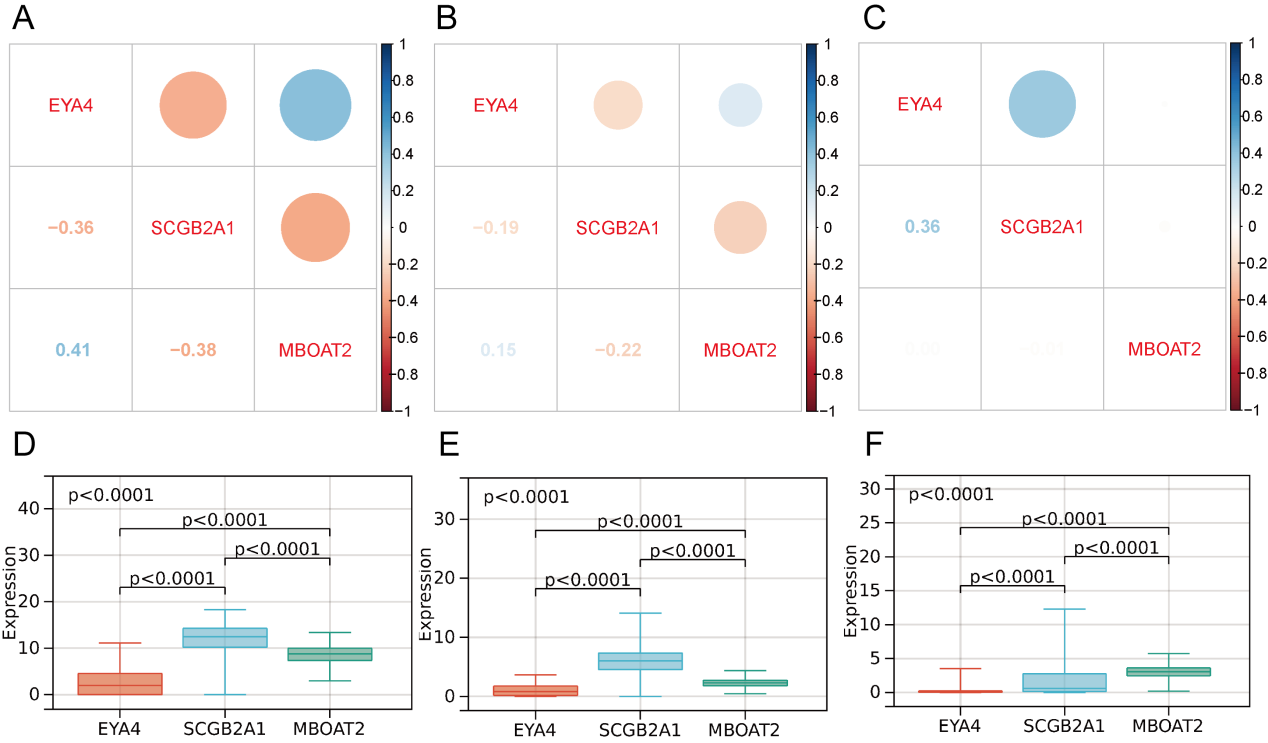


**Figure S3. Analysis of key gene correlations in three types of cancer. (A)** The key gene correlation analysis for endometrial cancer was conducted. **(B)** The key gene correlation analysis for ovarian cancer was conducted. **(C)** The key gene correlation analysis for cervical cancer was conducted. **(D)** The expression of key genes in endometrial cancer was examined. **(E)** The expression of key genes in ovarian cancer was examined. **(F)** The expression of key genes in cervical cancer was examined.


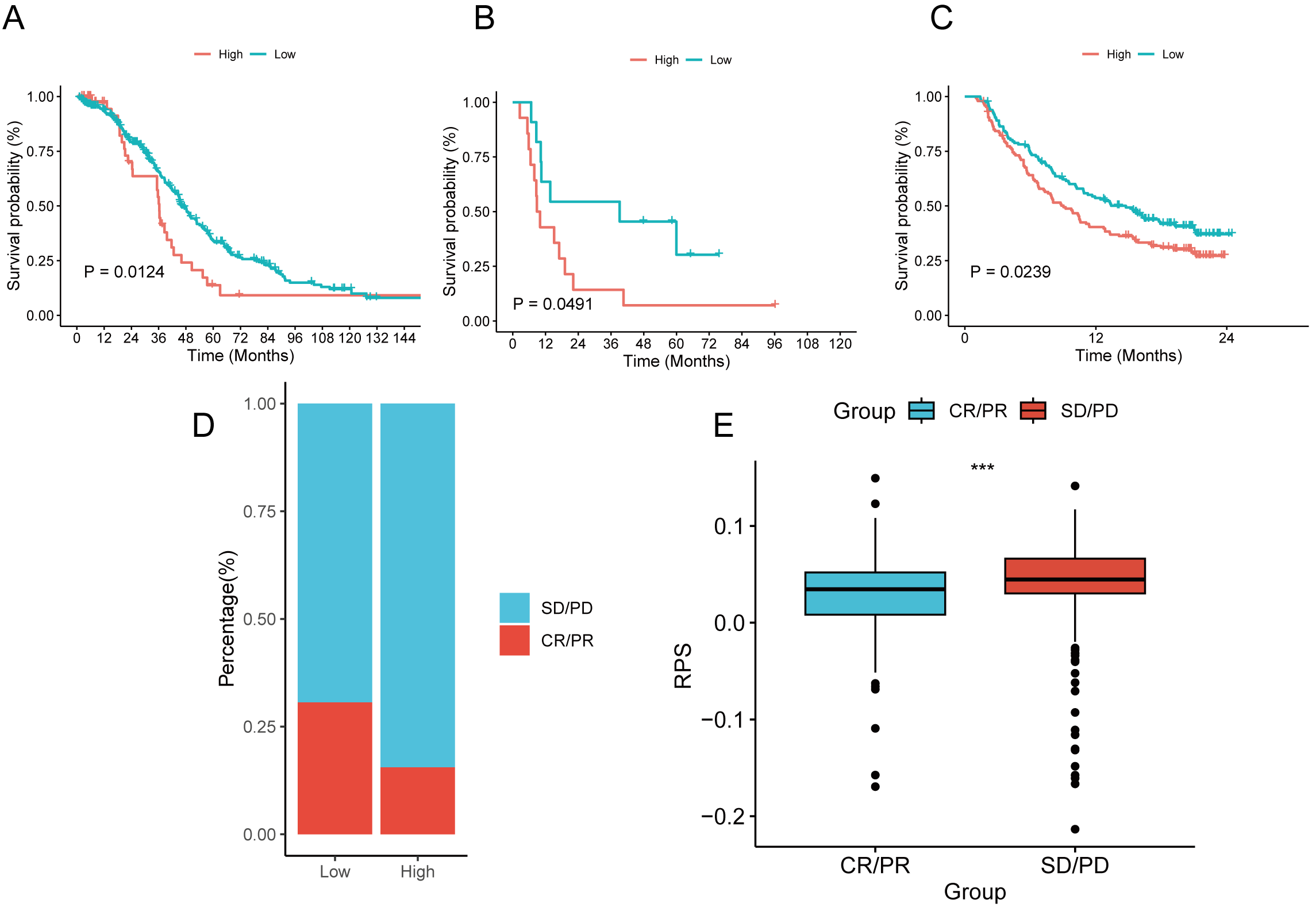


**Figure S4. Validation of RPS. (A)** OS Kaplan–Meier curves based on the high-RPS and low-RPS groups in TCGA-ovarian cancer cohorts. OS Kaplan-Meier curve of high-RPS and low-RPS groups population in immunization cohort GSE135222 **(B)** and IMvigor210 **(C)** Kaplan-Meier curve of patient OS.Box charts **(D)** and bar charts **(E)** illustrate the treatment response (CR/PR) and (SD/PD) in high-RPS and low-RPS groups in the completion IMvigor210 cohort. ***p < 0.001.


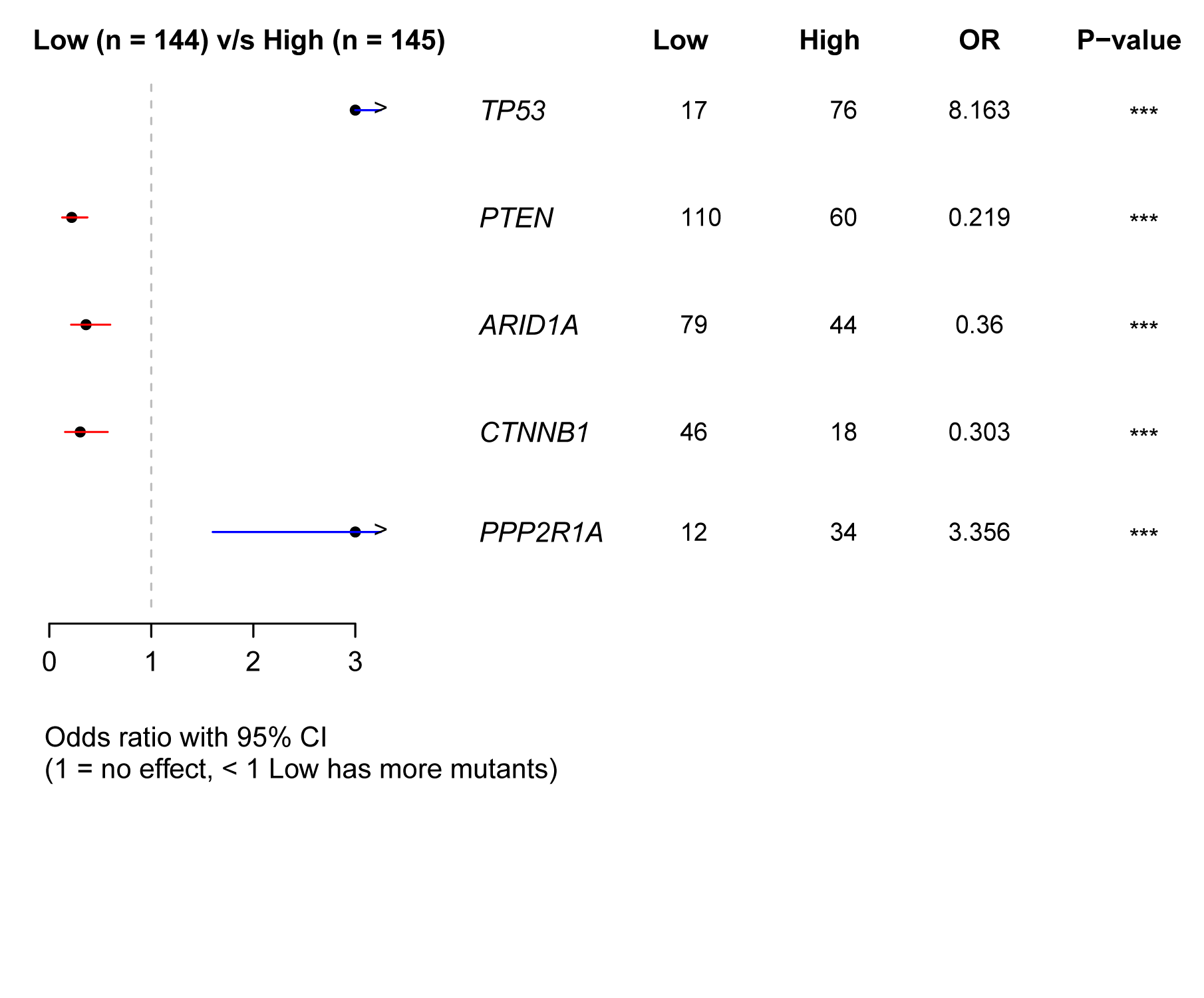


**Figure S5. Comparison of gene mutation frequency between high-RPS and low-RPS groups.**


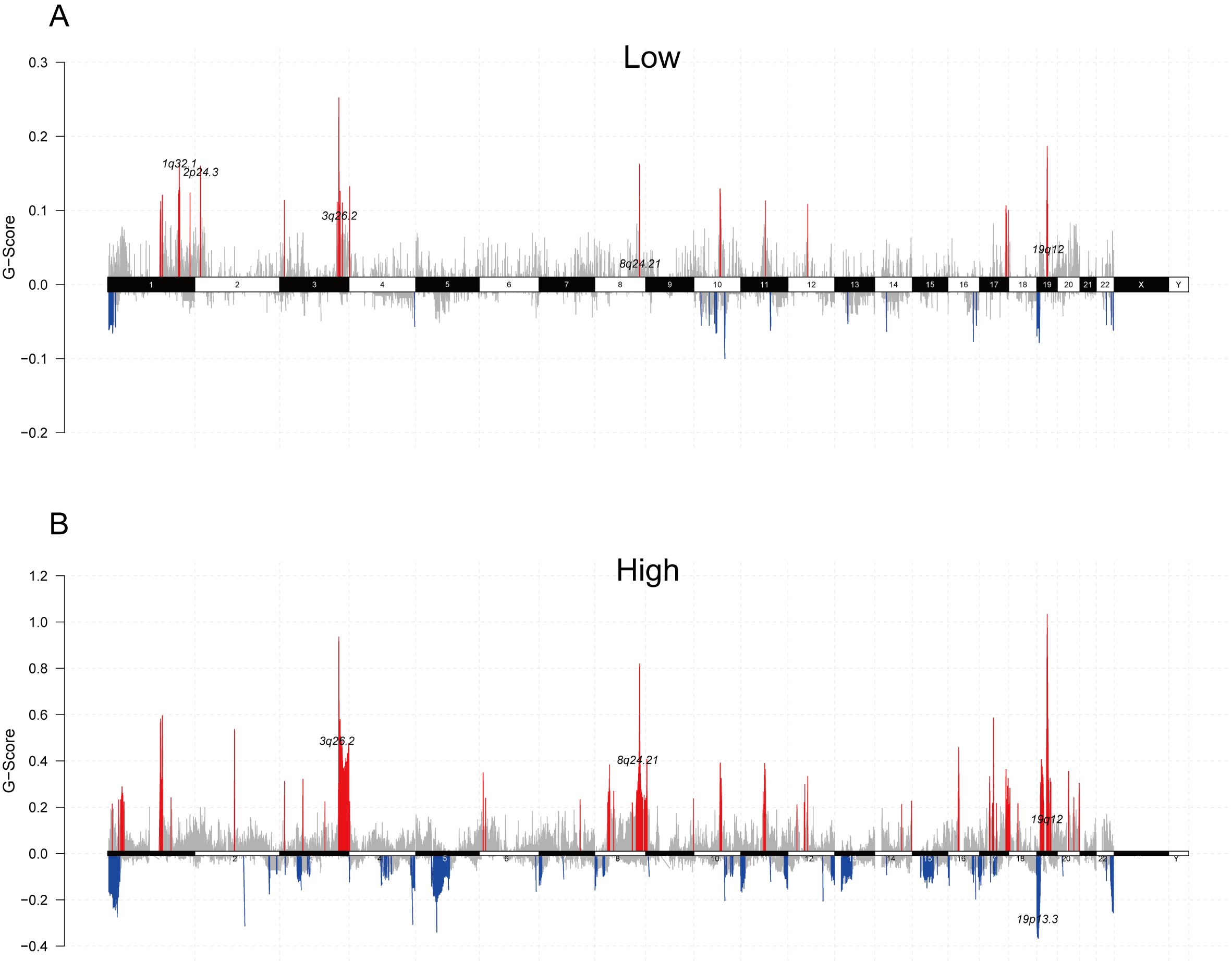


**Figure S6. Analysis of gene copy number variation.** Chromosome deletion region (blue) and amplification region (red) in low-RPS group **(A)** and high-RPS group **(B)**.


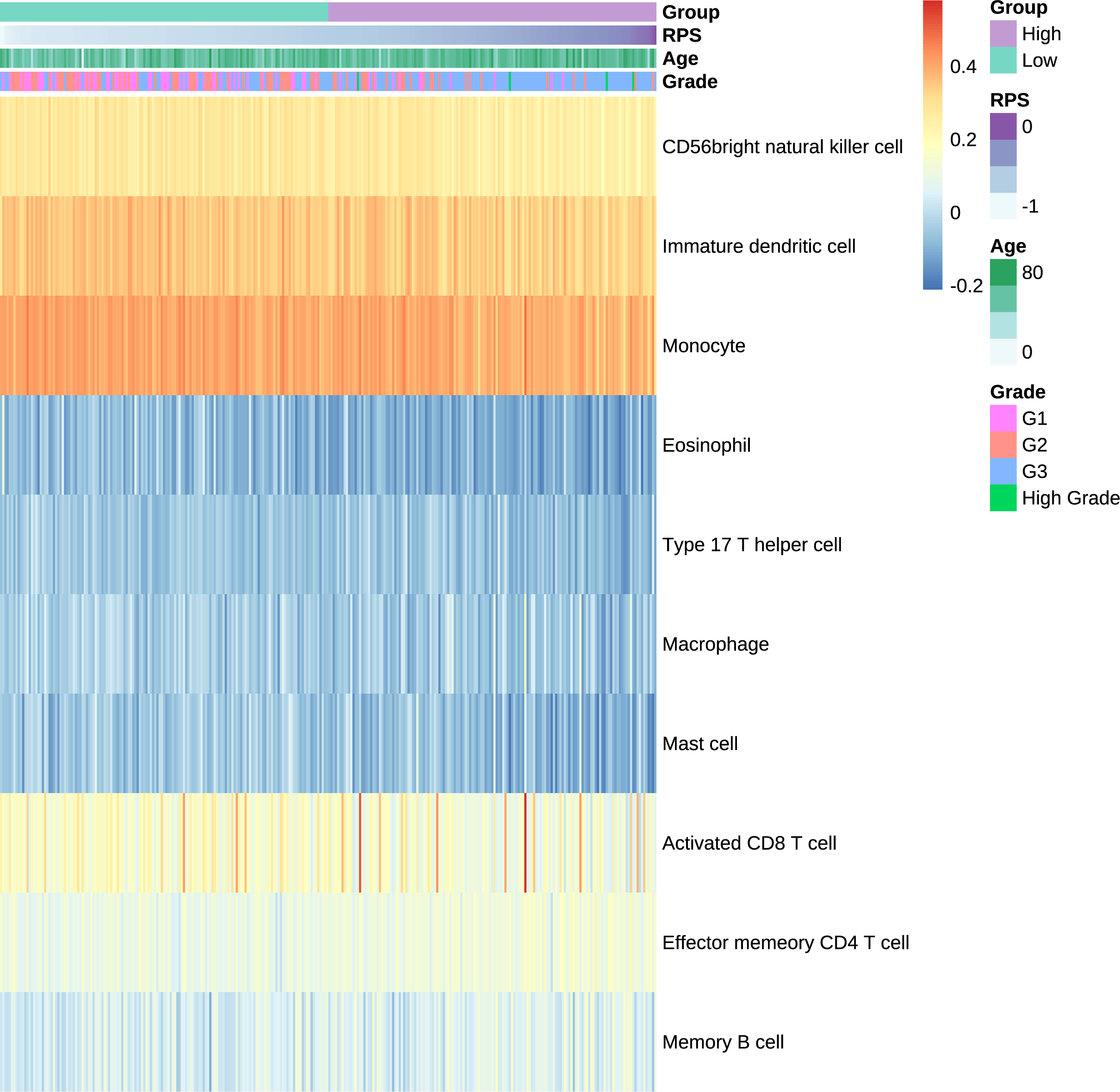


**Figure S7. Heatmap of the RPS relationship with the top 10 immune cells.**


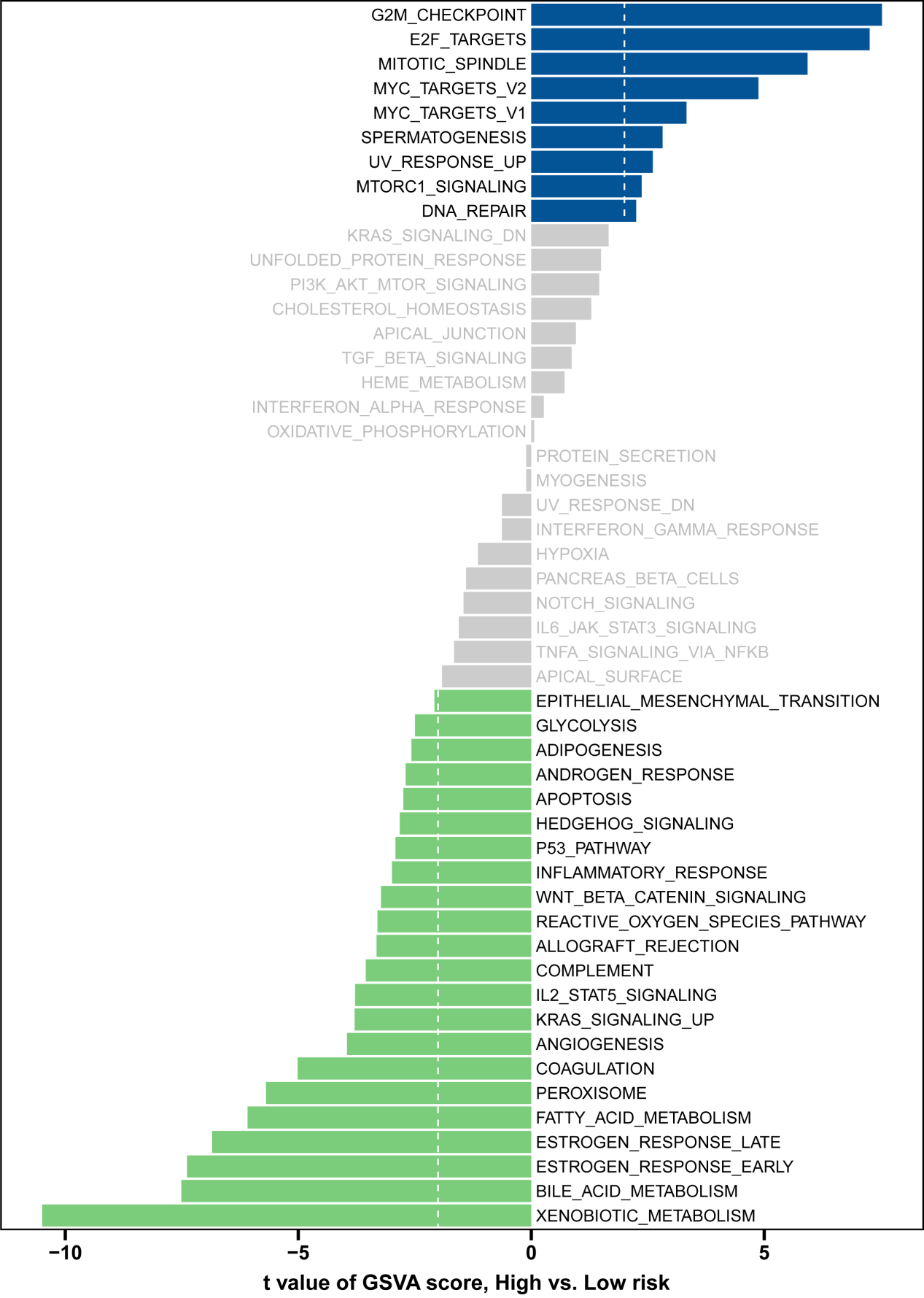


**Figure S8. The biological pathways had significant differences between high-RPS and low-RPS groups in GSVA.**


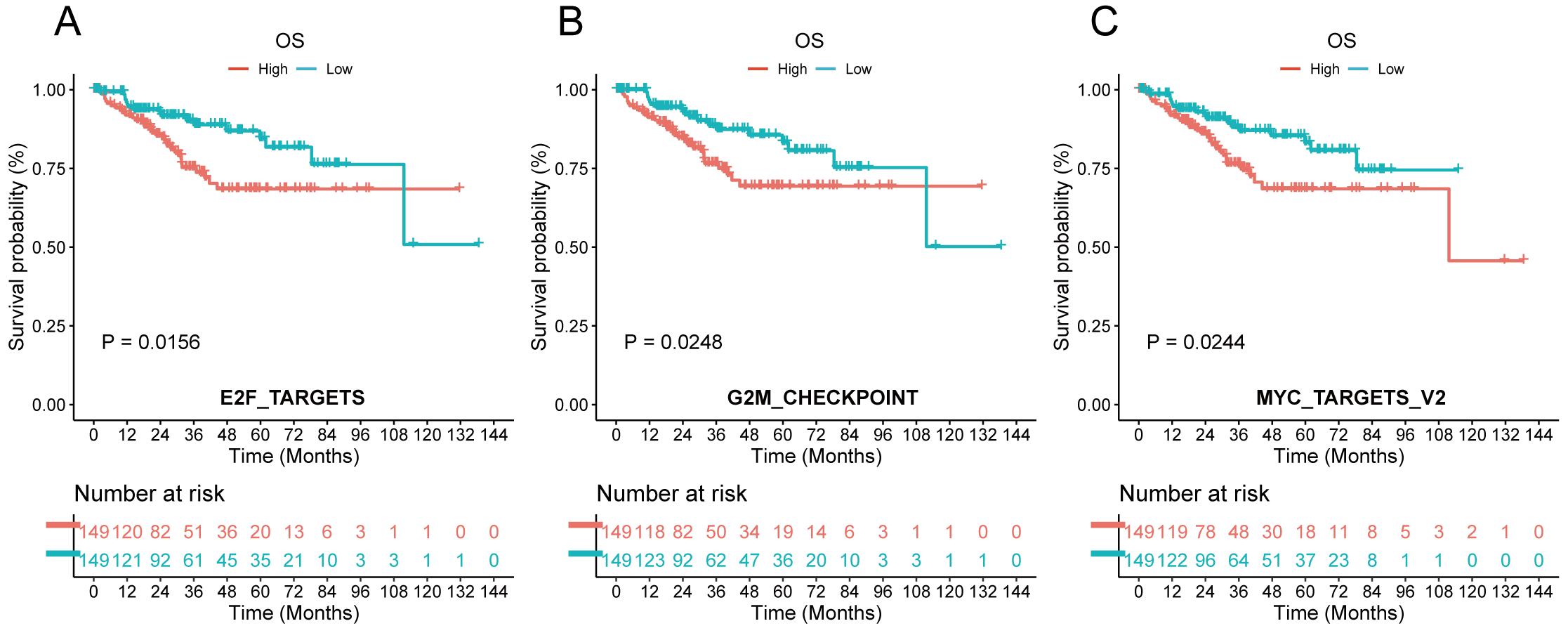


**Figure S9. Kaplan-Meier survival plots showing the significant correlations between the OS and GSVA scores of typical oncogenic hallmark pathways.**


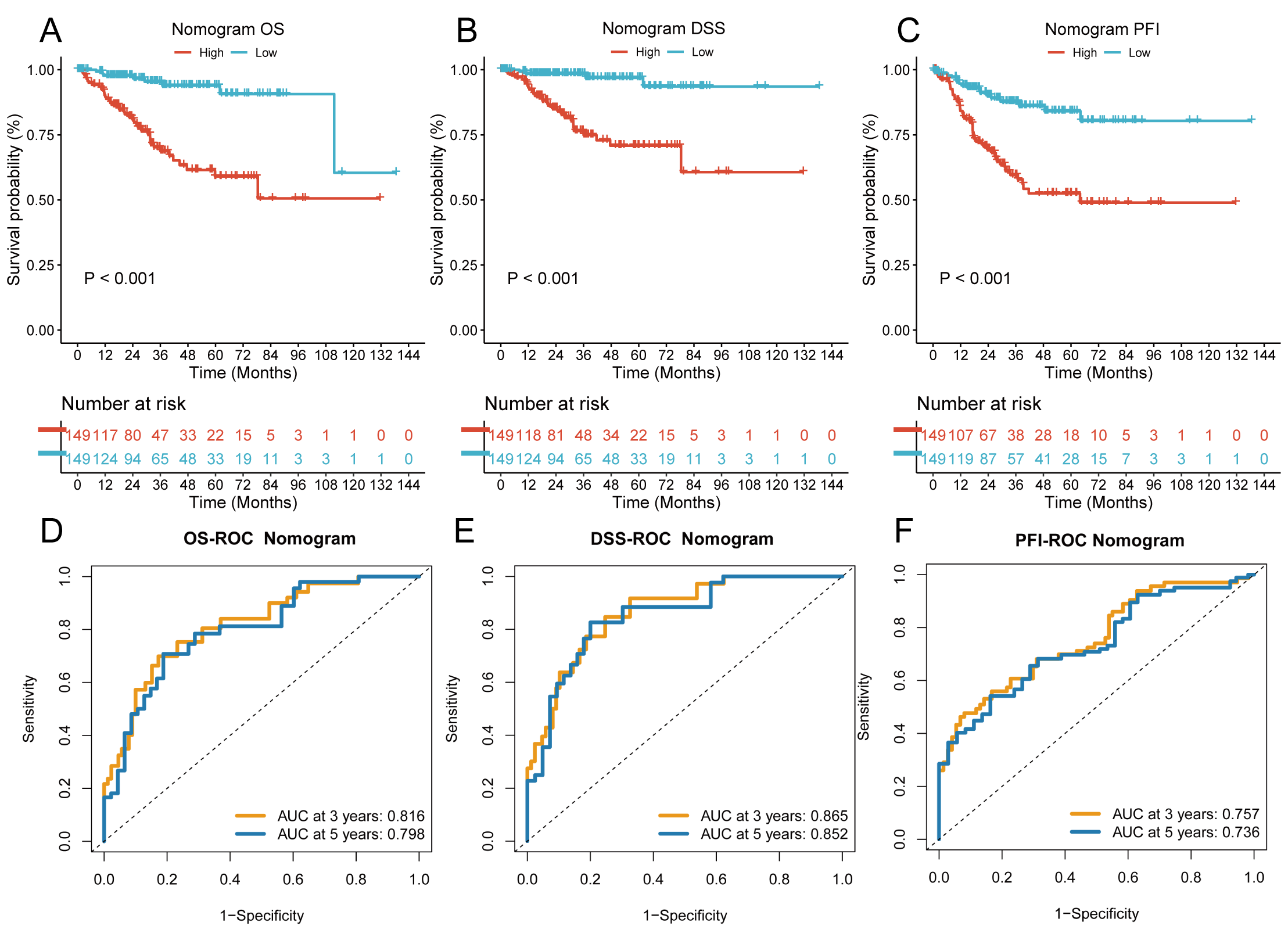


**Figure S10. Evaluation of the nomogram. (A-C)** Comparison of clinical outcomes between high-RPS and low-RPS groups using OS, DSS, and PFI as the endpoints. **(D-F)** ROC curves and AUC values of nomogram.

**Supplementary Table**

**Table S1.The characteristics of all included patients.**

| **Characteristics** | **level** | **non-Obesity** | **Obesity** | **P-value** |
| --- | --- | --- | --- | --- |
| **Total** |  | 205 | 298 |  |
| Age (mean (SD)) |  | 65.72 (11.96) | 62.26 (10.17) | 0.001 |
| Stage (%) | Stage1 | 125 (61.0) | 189 (63.4) | 0.645 |
|  | Stage2 | 17 ( 8.3) | 30 (10.1) |  |
|  | Stage3 | 52 (25.4) | 62 (20.8) |  |
|  | Stage4 | 11 ( 5.4) | 17 ( 5.7) |  |
| Diabetes (%) | Not Reported | 72 (35.1) | 93 (31.2) | <0.001 |
|  | NO | 112 (54.6) | 132 (44.3) |  |
|  | YES | 21 (10.2) | 73 (24.5) |  |
| hypertension (%) | Not Reported | 62 (30.2) | 78 (26.2) | 0.001 |
|  | NO | 77 (37.6) | 76 (25.5) |  |
|  | YES | 66 (32.2) | 144 (48.3) |  |
| Grade (%) | G1 | 35 (17.1) | 63 (21.1) | 0.046 |
|  | G2 | 37 (18.0) | 75 (25.2) |  |
|  | G3 | 126 (61.5) | 156 (52.3) |  |
|  | High Grade | 7 ( 3.4) | 4 ( 1.3) |  |

**Table S2.Baseline data of UCEC patients with obesity.**

| **Characteristics** | **level** | **High immune score group** | **Low immune score group** | **P-value** |
| --- | --- | --- | --- | --- |
| Total |  | 156 | 142 |  |
| Age (mean (SD)) |  | 61.35 (9.71) | 63.25 (10.59) | 0.107 |
| Stage (%) | Stage1 | 105 (67.3) | 84 (59.2) | 0.337 |
|  | Stage2 | 14 ( 9.0) | 16 (11.3) |  |
|  | Stage3 | 27 (17.3) | 35 (24.6) |  |
|  | Stage4 | 10 ( 6.4) | 7 ( 4.9) |  |
| Diabetes (%) | Not Reported | 46 (29.5) | 47 (33.1) | 0.38 |
|  | NO | 75 (48.1) | 57 (40.1) |  |
|  | YES | 35 (22.4) | 38 (26.8) |  |
| hypertension (%) | Not Reported | 41 (26.3) | 37 (26.1) | 0.978 |
|  | NO | 39 (25.0) | 37 (26.1) |  |
|  | YES | 76 (48.7) | 68 (47.9) |  |
| Grade (%) | G1 | 41 (26.3) | 22 (15.5) | 0.017 |
|  | G2 | 41 (26.3) | 34 (23.9) |  |
|  | G3 | 74 (47.4) | 82 (57.7) |  |
|  | High Grade | 0 ( 0.0) | 4 ( 2.8) |  |

**Table S3. Correlation between RPS and immune cell infiltration.**

| **symbol** | **Pearson correlation** | **P-value** |
| --- | --- | --- |
| Effector memory CD4 T cell | 3.55E-01 | 2.70E-10 |
| Memory B cell | 2.28E-01 | 7.21E-05 |
| Regulatory T cell | 2.02E-01 | 4.61E-04 |
| Activated CD4 T cell | 2.02E-01 | 4.61E-04 |
| Type 2 T helper cell | 1.54E-01 | 7.71E-03 |
| Natural killer T cell | 8.41E-02 | 1.47E-01 |
| Central memory CD8 T cell | 5.63E-02 | 3.33E-01 |
| Gamma delta T cell | 3.01E-02 | 6.05E-01 |
| Immature B cell | -3.94E-02 | 4.98E-01 |
| Type 1 T helper cell | -5.27E-02 | 3.64E-01 |
| Natural killer cell | -6.59E-02 | 2.56E-01 |
| T follicular helper cell | -7.45E-02 | 2.00E-01 |
| Activated B cell | -7.53E-02 | 1.95E-01 |
| Effector memory CD8 T cell | -1.21E-01 | 3.72E-02 |
| CD56dim natural killer cell | -1.38E-01 | 1.69E-02 |
| Neutrophil | -1.67E-01 | 3.93E-03 |
| Myeloid derived suppressor cell | -1.70E-01 | 3.25E-03 |
| Activated dendritic cell | -1.88E-01 | 1.08E-03 |
| Plasmacytoid dendritic cell | -2.00E-01 | 5.05E-04 |
| Macrophage | -2.05E-01 | 3.79E-04 |
| Central memory CD4 T cell | -2.11E-01 | 2.38E-04 |
| Activated CD8 T cell | -2.30E-01 | 6.00E-05 |
| Eosinophil | -2.75E-01 | 1.38E-06 |
| Immature dendritic cell | -2.86E-01 | 4.90E-07 |
| Monocyte | -2.98E-01 | 1.53E-07 |
| Type 17 T helper cell | -3.14E-01 | 3.16E-08 |
| CD56bright natural killer cell | -3.17E-01 | 2.23E-08 |
| Mast cell | -3.31E-01 | 4.75E-09 |

**Table S4. Univariate Cox regression analysis of RPS.**

| **symbol** | **HR** | **P-value** | **lower** | **upper** |
| --- | --- | --- | --- | --- |
| Age | 2.19E+00 | 2.27E-02 | 1.12E+00 | 4.30E+00 |
| Diabetes | 7.87E-01 | 4.95E-01 | 3.95E-01 | 1.57E+00 |
| Hypertension | 6.47E-01 | 3.00E-01 | 2.84E-01 | 1.47E+00 |
| Grade | 1.11E+01 | 2.26E-02 | 1.40E+00 | 8.76E+01 |
| Stage | 1.39E+00 | 5.55E-01 | 4.68E-01 | 4.11E+00 |
| RPS | 2.42E-01 | 6.75E-05 | 1.21E-01 | 4.87E-01 |

**Table S5. Correlation analysis among key genes in endometrial cancer.**

| Gene | EYA4 | SCGB2A1 | MBOAT2 |
| --- | --- | --- | --- |
| EYA4 | 1.00E+00 | -3.59E-01 | 4.10E-01 |
| SCGB2A1 | -3.59E-01 | 1.00E+00 | -3.80E-01 |
| MBOAT2 | 4.10E-01 | -3.80E-01 | 1.00E+00 |

**Table S6. Correlation analysis among key genes in ovarian cancer.**

| Gene | EYA4 | SCGB2A1 | MBOAT2 |
| --- | --- | --- | --- |
| EYA4 | 1.00E+00 | 3.61E-01 | 1.78E-03 |
| SCGB2A1 | 3.61E-01 | 1.00E+00 | -8.89E-03 |
| MBOAT2 | 1.78E-03 | -8.89E-03 | 1.00E+00 |

**Table S7. Correlation analysis among key genes in cervical cancer.**

| Gene | EYA4 | SCGB2A1 | MBOAT2 |
| --- | --- | --- | --- |
| EYA4 | 1.00E+00 | -1.89E-01 | 1.49E-01 |
| SCGB2A1 | -1.89E-01 | 1.00E+00 | -2.21E-01 |
| MBOAT2 | 1.49E-01 | -2.21E-01 | 1.00E+00 |
